# A Specific Ratio of Dietary Short-Chain and Long-Chain Fructo-Oligosaccharides Shifts the Immune Response Away from Type 2 in a Murine Model for House Dust Mite-Induced Asthma

**DOI:** 10.3390/nu17223520

**Published:** 2025-11-11

**Authors:** Roos E. M. Verstegen, Marit Zuurveld, Suzan Thijssen, Marjolein J. W. de Bruijn, Ingrid van Ark, Mara A. P. Diks, Johan Garssen, Gert Folkerts, Atanaska I. Kostadinova, Rudi W. Hendriks, Linette E. M. Willemsen

**Affiliations:** 1 Division of Pharmacology, Utrecht Institute for Pharmaceutical Sciences, Faculty of Science, Utrecht University, 3584 CG Utrecht, The Netherlands; m.zuurveld@uu.nl (M.Z.); s.thijssen@uu.nl (S.T.); i.vanark@uu.nl (I.v.A.); m.a.p.diks@uu.nl (M.A.P.D.); j.garssen@uu.nl (J.G.); g.folkerts@uu.nl (G.F.); 2 Department of Pulmonary Medicine, Erasmus Medical Center, University Medical Center, 3015 GD Rotterdam, The Netherlands; j.w.debruijn@erasmusmc.nl (M.J.W.d.B.); r.hendriks@erasmusmc.nl (R.W.H.); 3 Danone Research & Innovation, 3584 CT Utrecht, The Netherlands

**Keywords:** asthma, fructo-oligosaccharides, microbiome, short-chain fatty acids, house dust mite

## Abstract

**Background/Objectives**: The gut microbiome has an important role in immune regulation, and dietary interventions that support a balanced microbiota may help to prevent the development of allergic asthma. Dietary fibers can beneficially affect the intestinal microbiome, but due to the diversity of fiber types, the effects differ. In this study, we investigate the preventive effects of two mixes of short-chain and long-chain (1:1 and 9:1 ratio) fructo-oligosaccharides (FOS) in a mouse model of house dust mite (HDM)-induced allergic asthma. **Methods**: BALB/c mice received FOS-supplemented (1% *w*/*w*) diets before and during intranasal exposures to HDM. Endpoint airway hyperreactivity measurements were performed, followed by the collection of bronchoalveolar lavage fluid (BALF), lung, serum and cecum content. Fecal microbiome composition was determined by DNA sequencing and short-chain fatty acid (SCFA) levels were determined in the cecum, serum and lung. **Results**: Fecal microbiome analyses revealed an increased abundance of *Prevotellaceae* after FOS1:1 supplementation in HDM-allergic mice. Additionally, FOS1:1 protected against an HDM-induced increase in basal airway resistance. Both FOS1:1 and FOS9:1 restored the systemic acetate levels in HDM-allergic mice. The two FOS supplementations did not affect HDM-induced inflammatory cell influx in the BALF. However, FOS1:1 increased the frequency of Th1-cells and prevented an HDM-induced increase in the Th2/Th1 balance. Upon ex vivo restimulation with HDM, lung cell suspensions of FOS1:1-fed mice produced less type 2-related cytokines compared to control-supplemented mice, and FOS9:1 followed a similar pattern. **Conclusions**: Specific short-chain and long-chain FOS ratios differentially affect the microbiome and immune system in a mouse model with HDM-induced allergic airway inflammation. Dietary supplementation with FOS1:1 shifts the immune response away from type 2, suggesting that dietary fibers like FOS1:1 may contribute as a part of a broader strategy to modulate HDM-induced allergic asthma.

## 1. Introduction

The Global Asthma Network described that worldwide, 9.1% of children, 11% of adolescents and 6.6% of adults suffer from asthma symptoms, with some differences between regions [1]. There are different asthma endotypes, including type 2 high asthma involving sensitization to airborne allergens, such as house dust mites (HDMs) [2]. Allergic asthma is characterized by airway eosinophilia, chronic type 2 inflammation and increased airway hyperresponsiveness [2]. Environmental, lifestyle and dietary changes can increase the risk of allergy and asthma development [3].

Allergic asthma is associated with the atopic march, a concept that explains the development of allergic diseases. It starts at early infancy with atopic status and/or an allergic condition such as atopic dermatitis or food allergy, associated with an imbalance in type 2 over type 1 immunity [4]. This offers a window of opportunity at a young age for interventions that aim to prevent the development of allergic asthma. The gut microbiome plays an important role in immune regulation and can influence the health state of other organs^6^. The microbiome exerts these systemic effects mostly by fermenting fibers into metabolites, with the short-chain fatty acids (SCFAs) acetate, propionate and butyrate being the most abundant ones [5]. Especially butyrate is known to positively affect the immune system, since it has strong anti-inflammatory capacities and is known to support the development of regulatory T-cells [6]. At a young age, *Bifidobacterium* and *Lactobacillus* species are highly prevalent in the gut of breastfed infants [5], and are known to produce SCFA and cross-feed butyrate-producing microbes. Based on numerous studies showing the importance and flexibility of the early-life gut microbiome, dietary interventions that impact the microbiome are of increasing interest as preventative and or treatment opportunities [7].

Dietary fibers are generally reported to be beneficial for a healthy gut microbiome and can contribute to immune development [8]. Therefore, they are of interest as a strategy for allergy prevention. However, it must be taken into account that there is a large variety of fibers. In accordance with this diversity, they can affect the microbiome, SCFA production and other functional metabolites in dissimilar ways [8]. We previously presented a comprehensive review of many preventative studies with dietary fibers, both in mice and in humans, which reported very diverse results [8]. It is likely that the great diversity in fiber type and the dosage of fiber used in these studies (ranging from 1% to 30% *w*/*w*) profoundly influenced the observed outcomes.

A commonly used category of dietary fiber is the inulin-type fructans, which are well-known bifidogenic prebiotics. Two types of these fructans can be classified on the basis of their degree of polymerization (DP). There are short-chain (sc) fructo-oligosaccharide (FOS) variants with a DP of 2–10 and long-chain (lc) variants with a DP > 10, also known as inulin [9]. As a low DP is related to faster fermentation, sc- and lcFOS would differently affect SCFA production [10].

We previously showed that a dietary intervention with sc- and lcFOS (1:1) with *Bifidobacterium breve* (*B. breve*) beneficially impacted the immunological parameters in a murine model of HDM-induced allergic asthma [11]. However, this differs from the molecular size distribution of human milk oligosaccharides (HMOs), which is 9:1 [12]. The HMOs in human milk drive microbiome development and help protect against several diseases, including allergic asthma [13]. Prebiotics including FOS are added to formula milk to mimic some of the functional effects of HMOs [14]. As each fiber has unique effects on the microbiome and its fermentation processes [8], the immunomodulatory properties of FOS may depend on the molecular size distribution. In this study, the sc/lcFOS 9:1 and 1:1 ratio were compared in a murine model for acute HDM-induced allergic asthma, to elucidate the optimal usage of FOS in asthma prevention.

## 2. Materials and Methods

### 2.1. Diet Preparation

Experimental diets were based on an AIN93G semi-synthetic diet without glucose/lactose, in which casein was replaced by soy protein with added methionine and cysteine (ssniff-Spezialdiëten GMBH, Soest, Germany). scFOS (Raftilose P95, Beneo, Tienen, Belgium) and lcFOS (inulin HP, Cosucra, Warcoing, Belgium) were added to the diet in a 1:1 or 9:1 ratio (1% *w*/*w*). Supplementation with FOS was isocaloric and fibers were inter-exchanged with cellulose. Additional experimental groups were included, in which mice received *Bifidobacterium breve* (2 × 10^9^ CFU/g) (Morinaga Milk Industry, Tokyo, Japan) in their diet, either combined with the 1:1 or 9:1 diet, or not. Details of the dietary composition of all diets can be found in Appendix B (Table A3). Researchers were blinded when providing the diets to the mice.

### 2.2. Animals

A total of 36 seven-week-old male BALB/cAnNCrl mice (Charles River, Erkrath, Germany) were housed in individually ventilated cages with a maximum of 4 mice per cage. Housing conditions included a 12 h/12 h light/dark cycle (interventions performed during light phase), controlled temperature (21 ± 2 °C), controlled relative humidity (50–55%), woodchipped cage bedding, wood-curl nesting material, plastic orange shelter and a plastic orange tube. Mice had access to food and sterile water ad libitum. Upon arrival, mice were randomly assigned to the experimental groups and immediately received the intervention diets. The animals were allowed to acclimatize for two weeks, before starting the HDM exposure. This study was conducted under an ethical license provided by the national competent authority (CCD, Centrale Commissie Dierproeven) following positive advice from the Ethical Committee on the Use of Laboratory Animals of Utrecht University; all animal procedures were captured in protocols approved by the Animal Welfare Body, following the institutional Guidelines of the Ethical committee of Utrecht University—by this process securing full compliance with the European Directive 2010/63/EU for the use of animals for scientific purposes. Exclusion criteria were general disease symptoms, such as inactive behavior, deviant posture or >20% deviant weight compared to the average of the group, etc. No animals were excluded from this experiment.

Some outcome parameters of the sham and allergic mice fed the control diet will also be described in another publication [15].

### 2.3. Animal Procedures

On day 0, mice were sensitized intranasally (i.n.) with 40 µL PBS in the absence or presence of 5 µg HDM (02.01.86, Citeq, Groningen, The Netherlands), while under isoflurane anesthesia (induction 4% isoflurane, maintenance 1.5% isoflurane). On days 7–11, mice were challenged i.n. with PBS in the absence or presence of 15 µg HDM, while under isoflurane anesthesia. Feces were collected on day 11 after placing individual animals in a box for two minutes and taking the feces from the box. On day 14, mice were anesthetized by two i.p. injections of 150 µL saline solution containing a total of 4.92 mg ketamine (Narketanan^®^10, Vetoquinol, Magny-Vernios, France) and 0.033 mg Dexdormitor (Dexdormitor^®^, Vetoquinol), followed by airway resistance measurements and terminal cardiac puncture for blood collection. Additionally, bronchoalveolar lavage fluid (BALF), lung tissue and cecum content were collected. Further details on procedures can be found in Appendix A.

### 2.4. Airway Resistance

Under anesthesia, a small catheter was inserted in the trachea of the mice (to perform invasive airway resistance measurements). Mice were placed in a heated box to keep body temperature at 37 °C. The inserted catheter was connected to a pressure transducer (EMKA Technologies, Paris, France) mounted on the box. Mice were ventilated with a mixture of O_2_ and air (1:2) at a rate of 150 beats/min (tidal volume = 0.2 mL). Transpulmonary pressure was determined by measuring pressure differences in the catheter in the trachea. Airflow and tidal volume were measured using a flow transducer attached to the body box, detecting flow differences within the box. A saline solution, followed by increasing doses of methacholine (0–50 mg/mL, 10% puff for 10 s) were administered via aerosol from a nebulizer (EMKA Technologies) positioned between the mouse in the body box and the ventilator (EMKA Technologies). Pulmonary resistance (RL) was measured for 3 min following each methacholine dose. RL was calculated by dividing transpulmonary pressure by airflow at fixed points of constant long volume. The data are presented as average RL in cm H_2_O/(mL/s).

### 2.5. Bronchoalveolar Lavage Fluid (BALF)

Lungs were lavaged with 1 mL pyrogen-free saline (0.9% NaCl, 37 °C), containing protease inhibitor (Complete Mini, Roche, Basel, Switzerland). Consecutively, 3 lavages of 1 mL pyrogen-free saline (0.9% NaCl, 37 °C) were performed and pooled, followed by centrifugation to collect BALF cells (400× *g*, 5 min) and resuspended in 150 µL saline solution. Total numbers of BALF cells were counted in 20 µL BALF, using a Bürker–Türk chamber (magnification 100×).

### 2.6. Lung Cell Isolation

Lung tissue was enzymatically digested at 37 °C with digestion buffer (incl. DNase I and Collagenase A, Roche). Fetal calf serum (FCS, Cytiva, Marlborough, MA, USA) was added after 30 min to stop digestion. Lungs were processed using a 70 µm filter and rinsed with 10 mL RPMI 1640 culture medium (Lonza, Vacaville, CA, USA). After centrifugation (1400 rpm, 5 min, 4 °C), red blood cell lysis buffer (dH_2_O + 8.3 g/L NH_4_Cl + 1.0 g/L KHCO_3_ + 37.2 mg/L EDTA) was added for 4 min. To stop lysis, restimulation medium (RPMI 1640 culture medium + 10% FCS + 1% penicillin-streptomycin solution (Sigma-Aldrich, Burlington, MA, USA) + 0.02% 2-mercaptoethanol) was added. After centrifugation (1400 rpm, 5 min, 4 °C), cells were resuspended in restimulation medium.

### 2.7. Flow Cytometric Analysis of BALF and Lung Cells

General BALF immune cell differentiation was assessed using flow cytometry, as previously described by van Rijt et al. [16]. Lung single-cell suspensions were prepared as described earlier [17], and used for flow cytometry and ex vivo restimulation experiments.

Shortly, 60 µL BALF containing the cells of interest or 1 × 10^6^ of isolated lung cells was added to a plate and centrifuged for 5 min at 4 °C (14,000 RPM). Plates were washed with PBS, followed by the addition of Fixable Viability Dye (eFluor 780, Thermo Fisher Scientific, Waltham, MA, USA) for 30 min. Subsequently, plates were washed again and PBS blocking buffer containing 1% BSA, 5% FCS and 1% CD16/CD32 blocking solution was added for 10 min to block the non-specific background binding of antibodies and then washed off.

For BALF cells, antibodies against CD3e-PE-Cy7, MHCII-FITC, CD11c-APC, B220-Pe Cy7 (Thermo Fisher Scientific) and CCR3-PE (BioLegend, San Diego, CA, USA) in FACS buffer (1% Bovine Calf Serum in PBS) were added. Gate settings were based on ‘Fluorescence Minus One’ (FMO) staining controls or on previous work.

For the lung cell suspensions, two panels of extracellular antibodies were added. Panel 1 for Th1/Th2cell identification contained CD4-BV510 (BioLegend), CD69-PE-Cy7, CXCR3-PE (Thermo Fisher Scientific) and T1ST2-FITC (MDBioproducts, Zürich, Switzerland). Panel 2 for Th17/Treg cell identification contained CD4-BV510 (BioLegend), CD25-PerCP-Cy5.5, CD127-PE-Cy7 (Thermo Fisher Scientific) and CD196-PE (BioLegend). After fixation and permeabilization with Foxp3 Transcription Factor Staining Buffer Set (eBioscience, San Diego, CA, USA), cells were incubated with anti-FoxP3-FITC (Thermo Fisher Scientific) and anti-Rorγt-APC (BD Biosciences) (Panel 2).

Flow cytometry was conducted using FACS Canto II (BD Biosciences, San Jose, CA, USA) and analyzed using FlowLogic Software Version 1.0 (Inivai Technologies, Mentone, Victoria, Australia).

For specific ILC2 differentiation, 70 µL BALF was centrifuged for 7 min at 4 °C (400× *g*). The pellet was taken up in RPMI1640 (Gibco) + 10% FCS enriched with PMA (Merck, Darmstadt, Germany) + Ionomycin (Merck) and Golgistop (BD Biosciences) and cultured for 4 h at 37 °C and 5% CO_2_. After the incubation, the cells were washed with MACS buffer and stained with a lineage mix (consisting of PE-labeled antibodies to CD11b, CD11c, CD19, B220, NK1.1, GR-1, Ter119 and FceRIa), CD4-FITC, CD3-PE Texas Red, CD25-Pe Cy7, Sca-1-BV786, CD90.2-AF700 and CD8-APC eFluor780 and an Fc block and incubated for 30 min at 4 °C. After a PBS wash, a 15 min incubation with a Fixable Viability Dye was performed. Cells were washed with PBS, fixated with 2% PFA and permeabilized using 0.05% Saponin (Sigma-Aldrich) and stained with antibodies against IL9-PercP cy5.5, IL13-EF450, IFN-γ-BV650, IL4-BV711 and IL-5-APC, which were added for 1 h at 4 °C. Flow cytometry was conducted using FACS Symphony (BD Biosciences) and data were analyzed using FlowJo (v.10) (BD Biosciences).

More details on the used antibodies in this study can be found in the Appendix A.4.

### 2.8. Ex Vivo Lung Restimulation with House Dust Mite

Isolated lung cells (4 × 10^5^ cells/well) were cultured in RPMI1640 medium (+1% pen/strep + 10% FCS + 0.02% beta-mercaptoethanol) in the presence or absence of 25 µg/mL HDM. After 6 days of culture at 37 °C in 5% CO_2_, the supernatant was collected and stored at −20 °C until further analysis. Cell metabolic activity was determined using a WST-1 assay (Roche) according to the manufacturers protocol.

### 2.9. Preparation of Cecal Content

Weighted cecum content was diluted in 1 mL ice-cold PBS. Glass beads of size 1.0 mm (BioSpec Products, Bartlesville, OK, USA) were added, followed by 1.5 min vortexing. Homogenates were then centrifuged at 13,000 RPM for 10 min (4 °C). Collected supernatant was stored at −20 °C until further analysis.

### 2.10. Preparation of Serum

Blood samples were collected by cardiac puncture. The blood was coagulated for at least 30 min at room temperature before being centrifuged at 14,000 rpm for 10 min in MiniCollect^®^ serum tubes (Greiner Bio-One B.V., Alphen aan den Rijn, The Netherlands). Serum samples were stored at −20 °C until further use.

### 2.11. Preparation of Lung Homogenates

Lung samples that were stored at −80 °C were thawed and transferred to 2 mL tissue homogenizing CKMix tubes (Bertin Corp., Rockville, MD, USA), containing PBS with 1% Triton X100 (Sigma-Aldrich) and protease inhibitor (Complete Mini, Roche). Tissues were homogenized using a Precellys 24 tissue homogenizer (Bertin Corp.). Homogenates were centrifuged at 14,000 rpm for 10 min at 4 °C and supernatants were collected. Using the Pierce BCA protein assay kit, the protein concentration of each homogenate supernatant was determined, and standardized to BSA according to the manufacturer’s protocol (Thermo Fisher Scientific). Supernatants were stored at −20 °C until further use.

### 2.12. Chemokine, Cytokine and Antibody Measurements

Serum HDM-specific IgE was determined using an in-house-developed and validated protocol [17], using 1 µg/mL biotin anti-mouse IgE or 1 µg/mL biotin rat anti-mouse IgG1 (BD Pharmingen) detection antibodies. Serum concentrations of total IgE (BD Pharmingen, Franklin Lakes, NJ, USA), lung homogenate supernatant concentrations of IL-4, IL-5, IL-13, IFNγ, IL-10, CCL5 (Thermo Fisher Scientific), CCL20 and CCL22 (R&D systems, Minneapolis, MN, USA), and restimulation supernatant concentrations of IL-5 and IL-13 (Thermo Fisher Scientific) were measured according to the manufacturer’s protocol.

Plate read-outs were measured and analyzed using GloMax Discover (Promega, Madison, WI, USA). Concentrations of chemokines and cytokines in the lung homogenates were expressed as pg/mg protein, in restimulation supernatants and serum as pg/mL. HDM-specific IgE levels are expressed as the read-out value, optical density (OD).

### 2.13. Short-Chain Fatty Acid Quantification

Levels of acetate, propionate and butyrate were determined in 10 µL cecal content homogenate supernatant, serum and lung homogenate supernatant by LC-MS/MS. A detailed description of the methods can be found in Appendix A.2. Shortly, we used the Nexera X2 chromatographic system (Shimadzu, Kyoto, Japan) for liquid chromatography-tandem mass spectrometry (LC-MS) and the QTRAP^®^ 5500 triple quadrupole mass spectrometer (MS) (AB-SCIEX, Concord, ON, Canada). Internal standards included acetic acid-d4 (50 µM) (Thermo Fisher Scientific), propionic acid-d3 (20 µM) (Toronto Research Chemicals, Vaughan, ON, Canada) and butyric acid-d7 (10-µM) (Cayman Chemical, Ann Arbor, MI, USA).

SCFA concentrations of cecum content and lung were corrected for the initial tissue weight, by expressing the values of the SCFA concentration as mol SCFA per gram of tissue.

### 2.14. Fecal Microbiome Analysis

Fecal samples were sent to BGI Genomics (Hongkong), and microbiome composition was determined. Roughly 70 mg of feces was collected from mice on day 11. To obtain sufficient power for microbiome analyses, *N* = 5 per group was used for sample analyses. A detailed description of the methods can be found in Appendix A.3. Shannon’s index for alpha diversity was determined by BGI using Mothur (v.1.31.2) software. Beta diversity analysis (unweighted UniFrac) was performed by BGI using QIIME (v1.80) software. To correct for differences in sequencing depth, BGI normalized sequencing: sequences are extracted randomly according to the minimum sequence number for all samples, the extracted sequences formed a new ‘OTU table biom’ file, then the beta diversity distance was calculated based on the ‘OTU table biom’ file. Principal Coordinates Analyses (PCoA) were performed by BGI to visualize (dis)similarities of data using QIIME (v1.80). Differences in relative abundance of bacteria were visualized and analyzed on a family level. The top 10 most abundant families were taken into account; all other families were defined as ‘other’.

### 2.15. Statistical Analysis

The sample size of the experimental groups was determined by performing an ANOVA (fixed effects, omnibus, one-way) test with a priori power analysis (G*Power 3.1.9.2).

Results are presented as mean ± standard error of mean (SEM). Statistical analyses were conducted using GraphPad Prism software (version 10.4.1). The experimental unit was a single animal. Gaussian distribution was checked for all data. Data were transformed in the absence of a Gaussian distribution. For each outcome parameter, the allergic asthma model was validated by performing an unpaired *t*-test, a Welch’s *t*-test or Mann-Whitney test to compare the sham mice vs. HDM-allergic mice. Data of the dietary intervention groups were then analyzed using a one-way ANOVA, followed by a Dunnett’s multiple comparisons test, or by a Kruskal–Wallis test with Dunn’s multiple comparisons test, or by a Brown–Forsythe and Welch ANOVA with Dunnett’s T3 multiple comparisons test, testing all groups against the HDM-allergic control. Choice for which test to use depended on how well the data met the key assumptions of the unpaired *t*-test or one-way ANOVA. Two-tailed *p*-values were generated for all analyses, and *p* < 0.05 was considered significant. For fecal microbiome analyses, weighted UniFrac Adonis (PERMANOVA) testing was performed to test if microbiome communities of experimental groups differed from each other (QIIME (v1.80)).

## 3. Results

### 3.1. Dietary Intervention with FOS1:1, but Not FOS9:1, Increases Fecal Prevotellaceae Abundance, While Both FOS Interventions Increase Systemic Acetate in HDM-Allergic Mice

**Figure 1:**
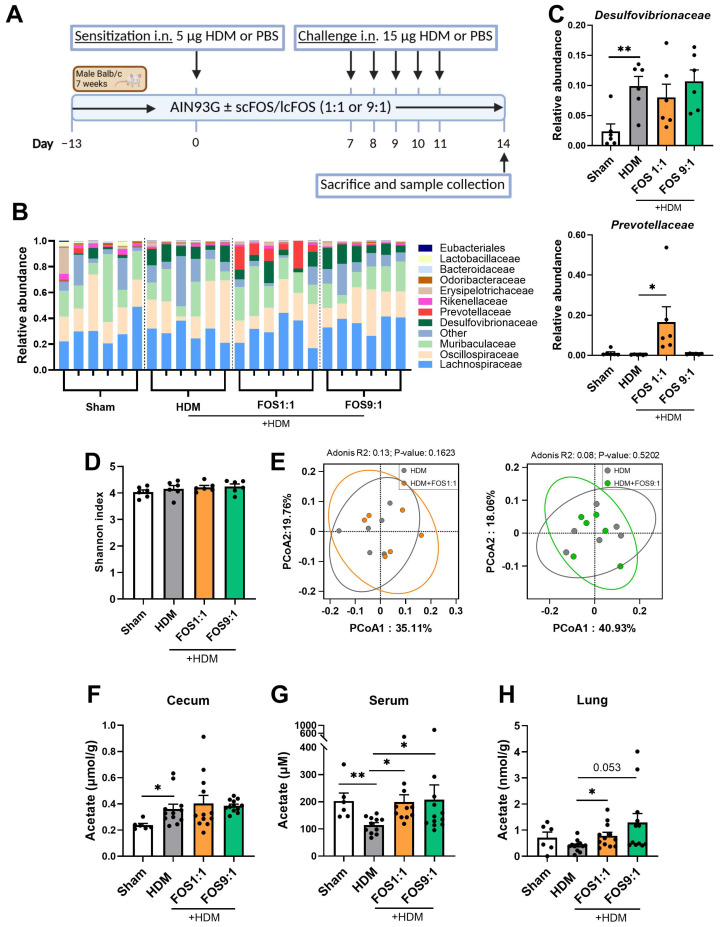
Effects of FOS1:1 and FOS9:1 on fecal microbiome composition and levels of acetate in cecum content, serum and lung in a mouse model for house dust mite allergic asthma. (**A**) Visual representation of the acute house dust mite (HDM) allergic asthma model in mice. (**B**–**E**) Microbiome composition was determined in feces of a random selection of mice out of each experimental group by DNA sequencing (*N* = 6 per group). Each dot in (**C**–**H**) represents one mouse. (**B**) Relative abundance of the top 10 bacterial families found in the fecal samples. (**C**) Relative abundance of the families Desulfovibrionaceae and Prevotellaceae compared between groups. (**D**) Alpha diversity as determined by Shannon index. (**E**) Principal Coordinates Analysis for beta diversity between HDM and HDM + FOS1:1 group, and between HDM and HDM + FOS9:1 group. (**F**–**H**) Acetate levels were quantified, using LC-MS/MS, in (**F**) cecum content homogenate supernatants, (**G**) serum and (**H**) lung homogenate supernatants. For fecal microbiome: *N* = 6. For SCFA: *N* = 6 for sham, *N* = 12 for HDM groups. In figures (**C**,**D**,**F**–**G**), sham and HDM groups were compared by an unpaired *t*-test. Effects of the diet (HDM vs. diet groups) were tested by one-way ANOVA or Brown–Forsythe and Welch ANOVA test with either Dunnett’s or Dunnett’s multiple comparisons tests. Results are shown as mean ± SEM (* *p* < 0.05, ** *p* < 0.01). In figure (**D**), an Adonis (PERMANOVA) test was performed to test whether microbial composition differed per experimental group.

To study the immune-modulatory effects of FOS, mice received an FOS1:1, FOS9:1 or control diet and were subjected to an HDM-induced model of allergic asthma (Figure 1A).

**Figure A1:**
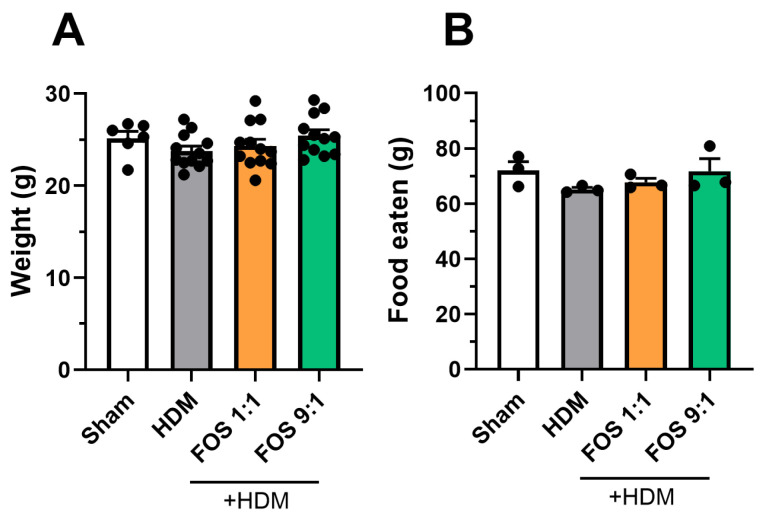
Background information animals. (**A**) Body weight of animals at end of the study (g). Each datapoint represents one animal. *N* = 12 (sham *N* = 6). (**B**) Average food eaten (g) per week per cage. Each datapoint represents the average of one cohort. N = 3. The model effect (sham vs. HDM) was tested using an unpaired *t*-test. Effects of the diets on food intake and body weight (HDM vs. HDM diet groups) were tested using one-way ANOVA followed by post hoc Dunnett’s multiple comparisons test. Results are shown as mean ± SEM.

**Figure A2:**
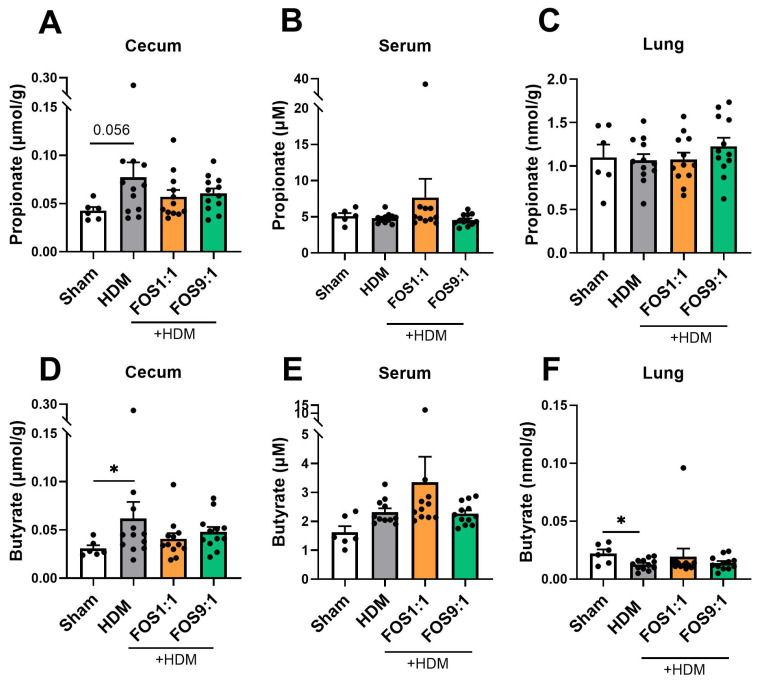
Propionate and butyrate levels in different tissue compartments, quantified using LC-MS/MS. Propionate and butyrate levels were determined in (**A**,**D**) cecum content homogenate supernatants, (**B**,**E**) serum and (**C**,**F**) lung homogenates. *N* = 6 for sham, *N* = 12 for HDM groups. Sham and HDM groups were compared by an unpaired *t*-test (with/without Welch’s correction) or Mann–Whitney test. Effects of the diet (HDM vs. diet groups) were tested by one-way ANOVA or Kruskal–Wallis test with either Dunnett’s or Dunn’s multiple comparisons tests. Results are shown as mean ± SEM. Each dot represents one mouse. (* *p* < 0.05).

Food intake and final body weight of the mice did not differ between the intervention groups (Figure A1). When we investigated fecal microbiome composition, changes were detected in the abundance of particular bacterial families in HDM-allergic mice compared to sham (Figure 1B). The microbiome of allergic mice was characterized by an increased abundance of *Desulfovibrionaceae* (Figure 1C), while the abundance of *Eubacteriales* (*p* = 0.0931) and *Rikenellaceae* (*p* = 0.0501) showed a decreasing trend as compared to sham. The FOS diets did not counteract these changes. Nevertheless, the FOS1:1 diet significantly enhanced the abundance of *Prevotellaceae* in the feces of HDM-allergic mice as compared to the control diet (Figure 1C), while the abundance of *Odoribacteriaceae* tended to decrease (*p* = 0.0565). Fecal microbiome sequencing did not reveal any effects of FOS1:1 or FOS9:1 on alpha diversity (species richness an evenness within individual mice) or beta diversity (dissimilarity between groups) in HDM-allergic mice (Figure 1D,E). SCFA levels were quantified in three different compartments, namely the cecum content, serum and lung. The acetate content in the cecum was increased in allergic mice compared to sham, but was not affected by the FOS1:1 or FOS9:1 diets (Figure 1F). In contrast, the acetate levels in the serum were significantly decreased in allergic mice compared to sham (Figure 1G). Both the FOS1:1 and FOS9:1 diets prevented this, and they also increased the acetate levels in the lungs of HDM-exposed mice (Figure 1H). The cecal propionate and butyrate quantities were higher in HDM-allergic mice than in sham mice (Figure A2A,D), while the serum concentrations remained unaffected (Figure A2B,E). In the lungs, the butyrate content declined in the HDM-allergic mice compared to sham (Figure A2F). The dietary interventions did not affect the propionate and butyrate levels in any of the measured compartments (Figure A2).

Taken together, these data show that in HDM-allergic mice, dietary intervention with FOS1:1, but not FOS9:1, affects the microbiome by increasing the abundance of fecal *Prevotellaceae*. Moreover, both FOS diet interventions increased the acetate content in the HDM-allergic mice.

### 3.2. Dietary Intervention with FOS1:1 Lowers Baseline Airway Resistance, but Does Not Affect Methacholine-Induced Airway Hyperreactivity

**Figure 2:**
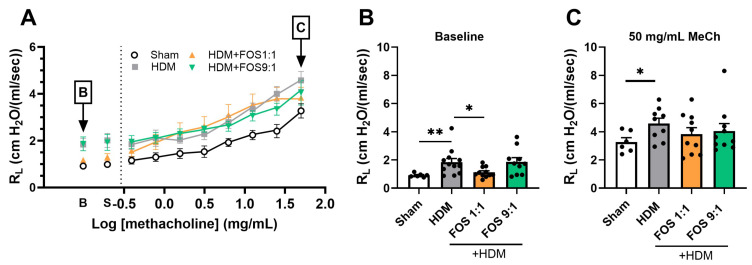
Effects of FOS1:1 and FOS9:1 on airway hyperreactivity. (**A**–**C**) FlexiVent invasive measurement of dynamic airway resistance in response to increasing doses of methacholine on day 14. Sham: PBS-sensitized and -challenged mice, + HDM: HDM-sensitized and -challenged mice. (**A**) Overview airway resistance at increasing doses of methacholine, log-scaled. B = baseline. S = saline solution. The vertical dotted line represents the change from baseline measurements to exposure to methacholine. (**B**) Airway resistance at baseline. (**C**) Airway resistance at exposure to 50 mg/mL methacholine. The experimental model (sham vs. HDM) was validated using an unpaired *t*-test (with or without Welch’s correction). Effects of the diets on allergy (HDM vs. HDM diet groups) were tested using one-way ANOVA followed by post hoc Dunnett’s multiple comparisons test. *N* = 12 (sham *N* = 6). Results are shown as mean ± SEM. In subfigure (**A**), each dot represents the average of the experimental group. In subfigure (**B**,**C**), each dot represents one mouse. (* *p* < 0.05, ** *p* < 0.01).

**Figure A3:**
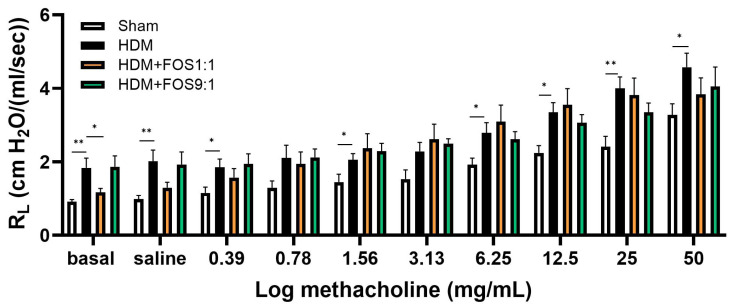
FlexiVent invasive measurement of dynamic airway resistance in response to increasing doses of methacholine on day 14. Overview airway resistance at increasing doses of methacholine (0.39–50 mg/mL). The model (sham vs. HDM) was validated using an unpaired *t*-test (with/without Welch’s correction) or Mann–Whitney test for each methacholine concentration. Effects of the diets on airway resistance (HDM vs. HDM diet groups) were tested using one-way ANOVA followed by post hoc Dunnett’s multiple comparisons test, again per methacholine concentration. *N* = 12 (sham *N* = 6). Results are shown as mean ± SEM (* *p* < 0.05, ** *p* < 0.01).

**Figure A4:**
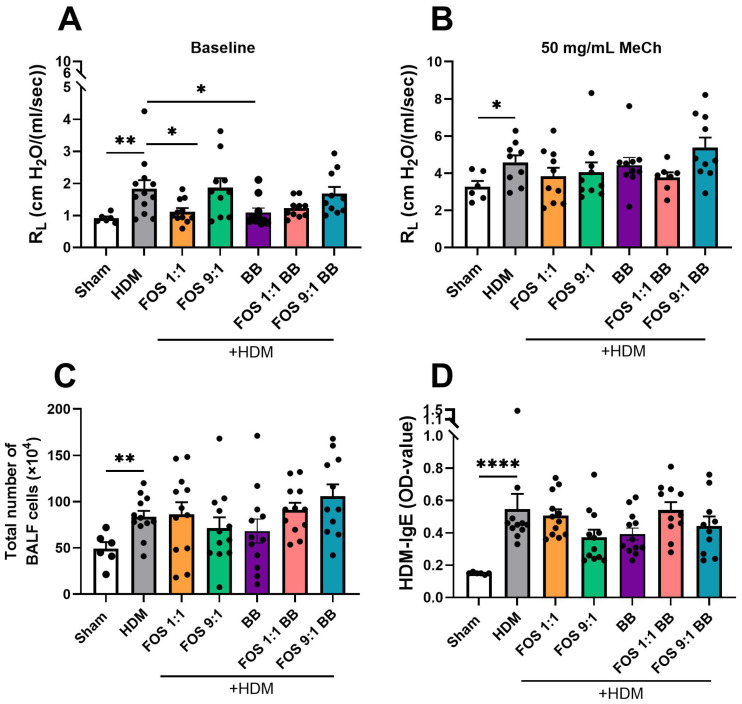
Effects of *Bifidobacterium breve* supplementation to FOS diets on airway hyperreactivity and HDM sensitization. FlexiVent invasive measurement of dynamic airway resistance in response to increasing doses of methacholine on day 14. (**A**) Airway resistance at baseline. (**B**) Airway resistance at exposure to 50 mg/mL methacholine. (**C**) Absolute number of inflammatory cell influx in BAL fluid. (**D**) HDM-specific IgE level in the serum. The model (sham vs. HDM) was validated using an unpaired *t*-test (with/without Welch’s correction) or Mann–Whitney test. Effects of the diets on allergy (HDM vs. HDM diet groups) were tested using one-way ANOVA or Brown–Forsythe and Welch ANOVA test followed by post hoc Dunnett’s (T3) multiple comparisons test. *N* = 12 (sham *N* = 6). Results are shown as mean ± SEM. Each dot represents one mouse. (* *p* < 0.05, ** *p* < 0.01, **** *p* < 0.0001).

Next, airway resistance in response to nebulized methacholine exposure was assessed, as a measure of lung function (Figure 2A). At baseline, airway resistance was significantly higher in HDM-allergic control mice compared to sham, while dietary intake of FOS1:1 mitigated this baseline increase (Figure 2B). Exposure to progressive doses of methacholine increased airway resistance in all groups (Figure 2A). In accordance with the findings at baseline, airway resistance was significantly higher in HDM-allergic control mice than in sham mice after almost all of these exposures (Figure A3; shown for 50 mg/mL in Figure 2C). However, when corrected for baseline resistance, allergic mice did not show an increase in airway hyperreactivity in response to methacholine when compared to sham. FOS diets did not affect the airway resistance in methacholine-exposed allergic mice at 50 mg/mL (Figure 2C), nor at any of the lower doses (Figure A3). Additional experimental groups, in which diets were supplemented with *B. breve* (with or without FOS), also did not affect airway hyperreactivity upon exposure to methacholine. Similar to FOS1:1, *B. breve* supplementation prevented an HDM-induced rise in basal resistance (Figure A4A,B).

In summary, whereas dietary intervention with FOS1:1, but not with FOS9:1, reduces the baseline values of airway resistance, it did not affect methacholine-induced airway hyperreactivity.

### 3.3. Dietary Intervention with FOS Does Not Prevent the Inflammatory Response to HDM Allergens

**Figure 3:**
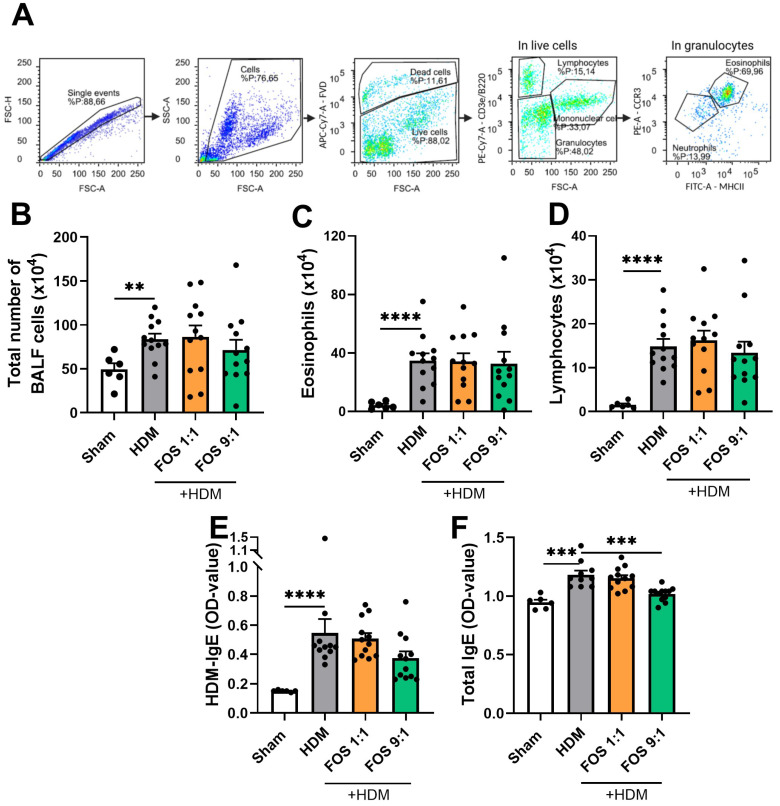
Inflammatory cell infiltration in bronchoalveolar lavage fluid (BALF) and HDM-induced allergic inflammation markers in serum. Sham: PBS-sensitized and -challenged mice, +HDM: HDM-sensitized and -challenged mice. (**A**) Representative gating strategy for flow cytometric analysis of cells obtained from BALF (eosinophils, neutrophils, lymphocytes, monocytes/macrophages). Numbers were calculated based on the percentage of cells derived from the flow cytometric analysis and the total BALF cell count using the Bürker–Türk counting room. (**B**) Absolute number of inflammatory cell influx in BAL fluid. (**C**) Number of eosinophils in BALF. (**D**) Number of lymphocytes in BALF. (**E**) HDM-specific IgE in serum and (**F**) total IgE in serum, expressed as OD value at 450 nm. The experimental model (sham vs. HDM) was validated using an unpaired *t*-test or Mann–Whitney test. Effects of the diets on allergy (HDM vs. HDM diet groups) were tested using one-way ANOVA or Kruskal–Wallis test followed by post hoc Dunnett’s or Dunn’s multiple comparisons test. *N* = 12 (sham *N* = 6). Results are shown as mean ± SEM. Each dot represents one mouse. (** *p* < 0.01, *** *p* < 0.001, **** *p* < 0.0001).

**Figure A5:**
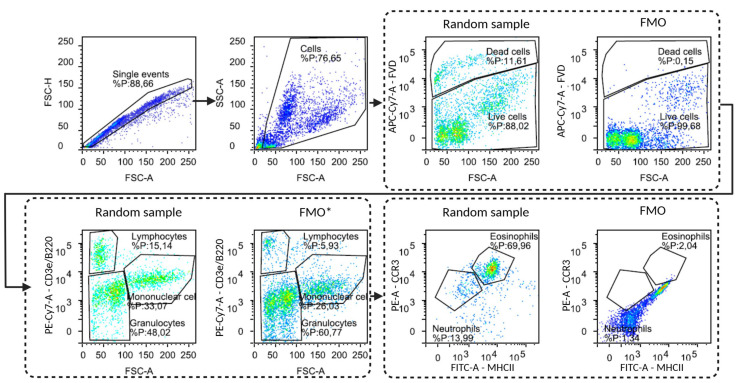
Gating strategy, including corresponding FMOs, for flow cytometric analysis of cells obtained from BALF (eosinophils, neutrophils, lymphocytes, monocytes/macrophages). Due to a practical error, the FMO* for the PE-Cy7 fluorochrome failed.

Next, airway inflammation was studied by assessing the inflammatory cell infiltration in the BALF using flow cytometry (see Figure 3A for basic gating strategy, Figure A5 for extended gating strategy). HDM-exposed mice showed a significantly higher total cell influx in the BALF than sham mice (Figure 3B). This increase was due to an influx of eosinophils (Figure 3C) and lymphocytes (Figure 3D), but not of neutrophils. Dietary interventions did not counteract these increases (Figure 3B–D).

As expected, HDM-induced allergic inflammation markers in the serum were assessed, to examine the dietary effects on the HDM-allergic sensitization and airway inflammation. HDM-exposed mice had significantly higher serum levels of HDM-specific and total IgE compared to sham mice (Figure 3E,F). Dietary interventions did not prevent the increase in HDM-specific IgE in the serum. However, the FOS9:1 diet did decrease the total IgE levels in HDM-allergic mice (Figure 3F). In additional experimental groups, in which diets were supplemented with B. breve, dietary intervention with FOS also did not affect inflammatory cell influx in the BALF nor the HDM-IgE levels in the serum (Figure A4C,D).

### 3.4. FOS1:1 Dietary Intervention Shifts the Th2/Th1-Cell Balance Away from Th2

**Figure 4:**
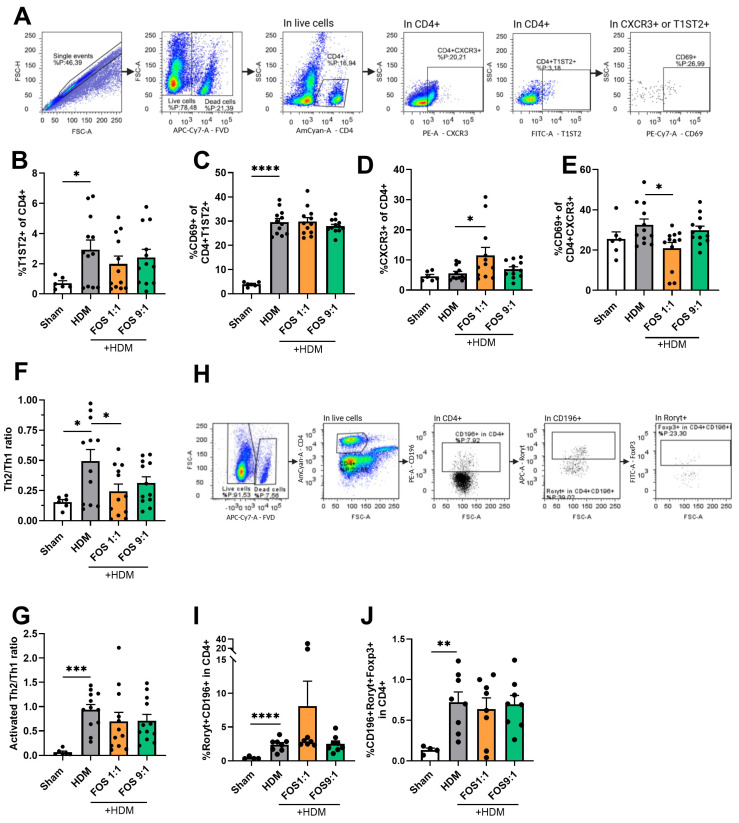
T-cell subsets in single-cell suspensions of the lung using flow cytometry. (**A**) Representative gating strategy for flow cytometric analysis of T-cells obtained from lung tissue (live/dead stain, CD4+, T1ST2+ (Th2), CXCR3+(Th1), CD69+ (activated T-cell). (**B**) %T1ST2+ cells of CD4+ live cells (Th2-cells). (**C**) Activated Th2 (CD69+ of CD4+T1ST2+). (**D**) %CXCR3+ cells of CD4+ live cells (Th1-cells). (**E**) Activated Th1 (CD69+ of CD4+CXCR3). (**F**) Ratio of Th2 vs. Th1-cells. (**G**) Ratio of activated Th2 vs. activated Th1 (%CD69+T1ST2+ of CD4+/%CD69+CXCR3+ of CD4+). (**H**) Representative gating strategy for flow cytometric analysis of T-cells from lung tissue (CD4+, CD196+, Rorγt+, Foxp3+). (**I**) % CD196+Rorγt+ cells of CD4+ live cells (Th17-cells). (**J**) CD196+Roryt+Foxp3+ cells of CD4+ live cells (regulatory Th17-cells). Mice from one cohort were excluded from the analyses of (**H**–**J**) (sham *N* = 4, HDM groups *N* = 8) due to a technical problem. The experimental model (sham vs. HDM) was validated using an unpaired *t*-test. Effects of the diets on allergy (HDM vs. HDM diet groups) were tested using one-way ANOVA followed by post hoc Dunnett’s multiple comparisons test. For (**B**–**E**): *N* = 12 (sham *N* = 6). For (**I**,**J**): *N* = 8 (sham *N* = 4). Results are shown as mean ± SEM. Each dot in subfigures (**B**–**J**) represent one mouse. (* *p* < 0.05, ** *p* < 0.01, *** *p* < 0.001, **** *p* < 0.0001).

**Figure A6:**
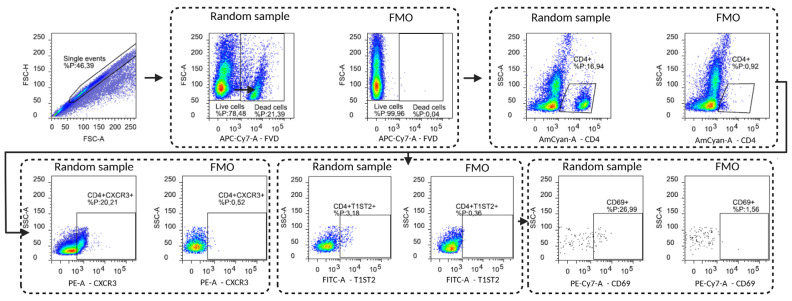
Gating strategy, including corresponding FMOs, for flow cytometric analysis of T-cells obtained from lung tissue. Live/dead stain, CD4+, T1ST2+ (Th2), CXCR3+(Th1), CD69+ (activated T-cell).

**Figure A7:**
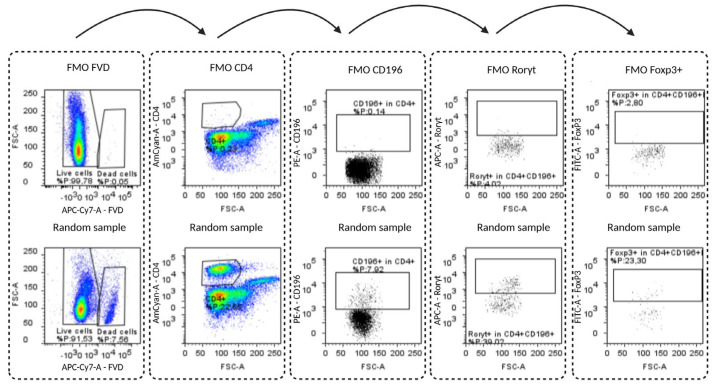
Representative gating strategy for flow cytometric analysis of T-cells from lung tissue. Live/dead stain, CD4+, CD196+, Rorγt+, Foxp3+).

To examine the inflammatory cells in the lungs in more detail, we performed flow cytometric analyses, focusing on T-cells (see Figure 4A,H for basic gating strategy, Figure A6 and Figure A7 for extended gating strategy). HDM-exposed mice had a significantly increased frequency of T1ST2+ cells within the CD4+ T-cell population, indicative for Th2-cells compared to sham mice (Figure 4B). Moreover, the proportion of CD69+ cells within the T1ST2+CD4+ T-cell fraction was also increased (Figure 4C). On the other hand, the proportions of CXCR3+ cells within the CD4+ T-cell population (Th1-cells) or their activation status (CD69+ fraction) remained unaffected (Figure 4D,E). Together, these findings indicate a Th2-skewed allergic inflammation, as reflected by an increased Th2/Th1 ratio of the total CD4+ cells or within the CD69+ fractions (Figure 4F,G).

The different dietary interventions did not affect the proportions of Th2-cells. However, when compared with the control diet, the FOS1:1 diet significantly increased the proportion of Th1-cells and prevented the HDM-induced increase in the Th2/Th1 ratio in allergic mice (Figure 4B–F). A similar pattern was observed for the Th2/Th1 ratio of activated CD69+ T-cells, but this did not gain significance (Figure 4G).

Additionally, the proportions of Th17-cells (positive for CCR6/CD196 and Rorγt) and regulatory Th17-cells (positive for CCR6/CD196, Rorγt and Foxp3) within the CD4+ T-cell population were increased in HDM-allergic mice fed the control diet compared to sham animals. FOS diet interventions did not affect this increase (Figure 4I,J).

**Figure A8:**
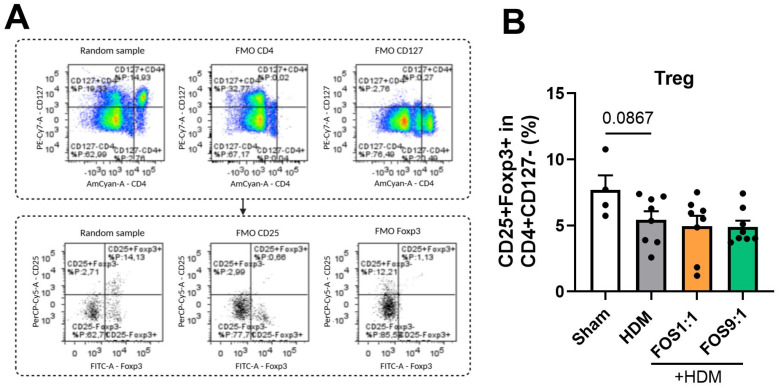
Regulatory T-cells in isolated lung cell suspensions. (**A**) Representative gating strategy for regulatory T-cells (CD4+CD25+CD127-Foxp3+). Strategy includes corresponding FMOs. (**B**) Treg cells. The model (sham vs. HDM) was validated using an unpaired *t*-test. Effects of the diets (HDM vs. HDM diet groups) were tested using one-way ANOVA followed by post hoc Dunnett’s test for multiple comparisons. *N* = 8 (sham *N* = 4). Results are shown as mean ± SEM. Each dot represents one mouse.

**Figure A9:**
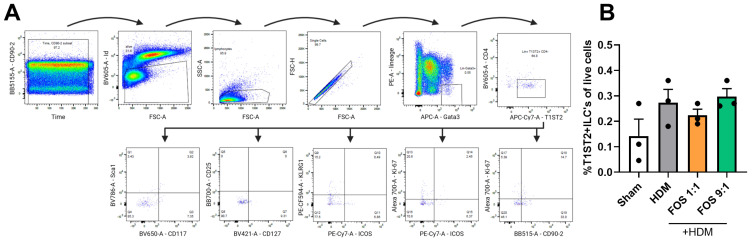
ILC2 levels in the BALF collected at day 14 as determined by flow cytometric analyses. (**A**) Representative gating strategy for the identification of ILC2s. (**B**) ILC2 levels (T1ST2 + ILCs) expressed as proportions of total live cells. The model (sham vs. HDM) was validated using an unpaired *t*-test. Effects of the diets on allergy (HDM vs. HDM-diet groups) were tested using one-way ANOVA followed by post hoc Dunnett’s multiple comparisons test. Because of the low numbers of ILC2s present, the values *N* = 3 represent data that were pooled from 4 mice. Results are shown as mean ± SEM. Each dot represents one mouse.

Furthermore, the frequencies of total lung CD25+Foxp3+ Treg were not affected by HDM exposure or the FOS diet interventions (Figure A8). Likewise, the frequencies of group 2 innate lymphoid cells (ILC2s) in the BALF were also not significantly affected by HDM exposure or the FOS diet interventions (Figure A9).

**Figure A10:**
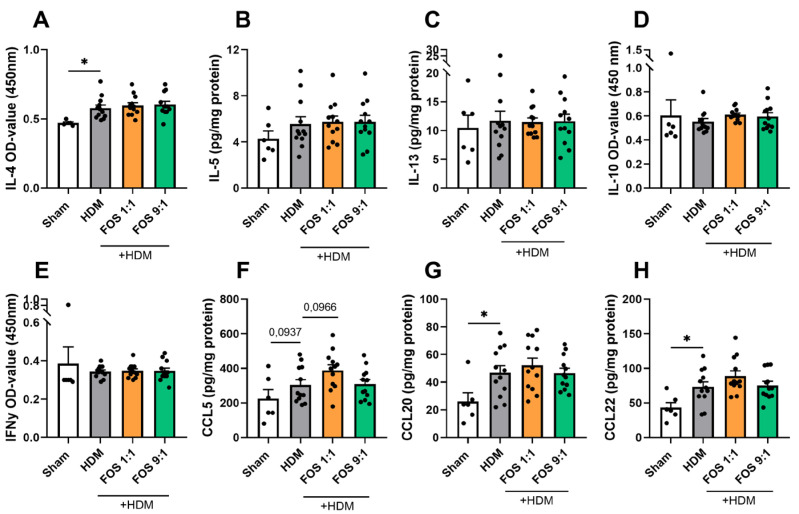
Airway inflammation-related cytokines and chemokines in lung tissue collected at day 14. Cytokine/chemokine levels were corrected for total protein levels in the lung, determined with a BCA assay. (**A**) IL-4 levels (OD value, (**B**) IL-5, (**C**) IL-13, (**D**) IL-10 (OD value), (**E**) IFNy (OD value), (**F**) CCL5, (**G**) CCL20 and (**H**) CCL22. The model (sham vs. HDM) was validated using an unpaired *t*-test or Mann–Whitney test. Effects of the diets on allergy (HDM vs. HDM diet groups) were tested using one-way ANOVA, Kruskal–Wallis or Brown–Forsythe and Welch ANOVA followed by post hoc Dunnett’s (T3) or Dunn’s multiple comparisons test. *N* = 12 (sham *N* = 6). Results are shown as mean ± SEM. Each dot represents one mouse. (* *p* < 0.05).

Finally, to further investigate the shifts in the Th2/Th1 balance, we measured the concentrations of type 2 inflammatory cytokines and chemokines in lung tissue homogenates. Although the IL-4, CCL20 and CCL22 concentrations were increased in HDM-exposed mice, these levels were not affected by the FOS diets (Figure A10).

Taken together, these findings indicate that FOS1:1 dietary intervention shifts the Th2/Th1-cell balance away from Th2 in the lungs of mice subjected to HDM-driven allergic airway inflammation.

### 3.5. In Vivo Dietary FOS Intervention Reduces Type 2 Cytokine Secretion upon Ex Vivo HDM Restimulation of Lung Cell Suspensions

**Figure 5:**
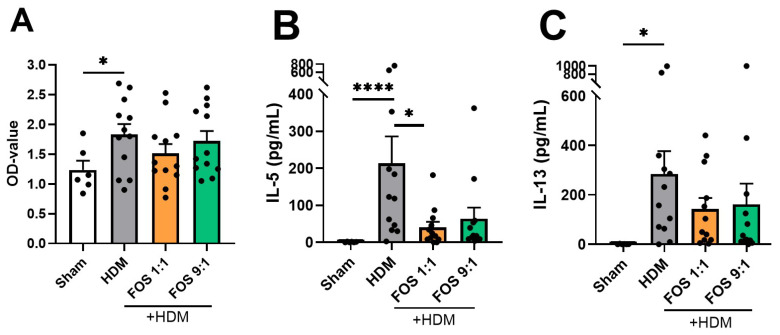
Cell response upon ex vivo HDM (25 µg/mL) restimulation of lung cells. (**A**) Metabolic activity of lung cells in medium with HDM, measured two hours after the start of the WST-1 assay. Increased OD values indicate increased metabolic activity. Supernatant concentrations of (**B**) IL-5 and (**C**) IL-13. The experimental model (sham vs. HDM) was validated using an unpaired *t*-test (with/without Welch’s correction). Effects of the diets on allergy (HDM vs. HDM diet groups) were tested using one-way ANOVA or Kruskal–Wallis test followed by post hoc Dunnett’s or Dunn’s multiple comparisons test. *N* = 12 (sham *N* = 6). Results are shown as mean ± SEM. Each dot represents one mouse. (* *p* < 0.05, **** *p* < 0.0001).

Finally, lung single-cell suspensions were ex vivo restimulated with PBS or HDM, to study allergen-specific T-cell responses. Cell metabolic activity was checked using a WST-1 assay, which quantifies the activity of mitochondrial dehydrogenases, at the end of the experiment (6 days). Cells derived from HDM-allergic mice fed the control diet were metabolically more active than cells derived from sham mice upon HDM stimulation (Figure 5A). In vivo FOS diet intervention did not affect the metabolic activity of the lung cell cultures. Upon restimulation of the lung single-cell suspensions, the PBS-exposed cells of HDM-allergic mice did not secrete detectable levels of type 2 cytokines (<10 pg/mL). Ex vivo restimulation with HDM induced significant IL-5 and IL-13 secretion in lung cell suspensions from mice that were fed the control diet (Figure 5B,C). These cytokines were not significantly induced in cell suspensions from mice that were fed FOS1:1 or FOS9:1 diets. In particular, compared with HDM-allergic mice fed the control diet, HDM-allergic mice fed FOS1:1 showed significantly reduced IL-5 production by lung cells upon HDM restimulation. Additionally, the IFNy, IL-10 and TNFα levels were measured, but in the restimulation cultures, the values of these cytokines were below the detection limit.

In summary, these findings show that dietary FOS intervention in mice subjected to HDM-driven allergic airway inflammation leads to a reduction in IL-5 and IL-13 production by lung single-cell suspensions upon ex vivo HDM restimulation.

## 4. Discussion

Dietary fibers beneficially affect the microbiome and can also directly regulate the immune system [7]. Due to the heterogeneity of fibers, the results of fiber interventions in allergic asthma studies are highly diverse [8]. In this study, we compared the effects of 1:1 and 9:1 ratios of sc- and lcFOS diets in an acute murine model of HDM-allergic airway inflammation.

Our findings show that an FOS1:1 diet alters the fecal microbiome composition of HDM-allergic mice by increasing the abundance of *Prevotellaceae*. Although the cecal SCFA levels were not affected by the FOS diets, both FOS1:1- and FOS9:1-fed HDM-allergic mice had increased acetate levels in the serum and lung. Regarding airway function, FOS1:1 prevented HDM-induced baseline airway resistance, but did not affect the increased airway resistance when HDM-allergic mice were exposed to methacholine. Additionally, the inflammatory cell influx in the BALF was not significantly modulated by the dietary interventions used. However, the FOS1:1 diet shifted the Th2/Th1 balance in HDM-allergic mice away from Th2, mainly by increasing Th1 frequency in the lung. Finally, FOS diets dampened type 2 cytokine production upon the ex vivo HDM restimulation of lung cell suspensions.

Previously, we have shown that HDM-induced allergic inflammation in mice already influenced the intestinal microbiome and SCFA profiles. We observed both an increase in the fecal abundance of *Desulfovibrionaceae* and a decrease in the serum and lung acetate levels in HDM-allergic mice (of note, data contained samples of control and allergic mice of the current study combined with a second study) [15]. The underlying causes of these effects are not yet unraveled. In this study, we focused on the effects of the different sc/lc FOS ratios of the dietary interventions on the intestinal microbiome and allergic asthma outcomes in this HDM allergy model. We observed the FOS1:1 and FOS9:1 ratio had divergent effects on the microbiome composition of allergic mice. These divergent effects, attributed to the different degrees in polymerization of the FOS, were previously described in various inflammatory disease models [18,19,20]. Research so far has focused on explaining the differences based on the indirect effects of the fibers, such as growth of and reduction in specific bacterial species. Whereas scFOS has been reported to stimulate *Bifidobacteria* [10,21], *Akkermansia* [10], *Lactobacillus* [10,21] and *Enterococcus* species [21], lcFOS was described to promote bacteria of the Clostridium coccoides–Eubacterium rectale cluster [21]. The abundance of these bacterial species was not significantly altered by the dietary interventions in our study. The literature shows that the abundance of bacteria throughout the murine intestinal tract, including the different regions of the colon, differs per region [22]. It could be hypothesized that *Prevotellaceae* species are less abundant in the proximal colon and more prevalent in the distal colon. As scFOS is rapidly fermented, this might happen predominantly in the proximal colon. lcFOS might reach the distal regions in higher amounts, thereby being fermented by *Prevotellaceae* and simultaneously stimulating the abundance of this bacterial species. However, as the current study measured the microbiome in fecal samples, we cannot distinguish the effects on the different regions of the intestinal tracts.

In addition to the indirect effects via the microbiota, direct effects of the fibers on cells, e.g., by binding directly to pattern recognition receptors on immune cells or epithelial cells, gained attention [23,24]. In vitro studies revealed that the differences in the chain length of β2 → 1-fructans, including FOS and inulin, influence the binding capacity and patterns to different Toll-like receptors (TLRs) [24]. The implications of this phenomenon appear to be complex. Sc fructans have shown weaker binding capacity to TLRs, but induced an anti-inflammatory cytokine pattern and intestinal barrier protecting effect in vitro, which was not observed for long-chain variants [23,24]. On the other hand, long-chain fructans were more successful in enhancing the immune response to hepatitis B vaccination and increasing Th1-cell differentiation, compared to sc variants [20]. The researchers hypothesized that these distinct effects could be attributed to differences in receptor interactions at the cell surface. Long-chain variants are larger and may therefore activate multiple neighboring TLR(-associated) receptors simultaneously, leading to synergistic downstream signaling [20].

In this study, FOS1:1, but not FOS9:1, induced a significant increase in the abundance of *Prevotellaceae*. Prevotella species in the gut have been strongly linked to carbohydrate-rich diets [25], and are potent fermenters of FOS [26]. Increased intestinal abundance of Prevotella genera was beneficially associated with offspring food allergy risk [27], but also with unfavorable immune responses [28,29]. In general, the role of Prevotella in human disease depends on the host population, the exact immunological aspects and the specific Prevotella strain [30]. For example, increased abundance of the genus Prevotella copri was beneficially associated with allergic diseases, including asthma [27,31,32,33]. However, recent work on the early-life gut microbiome in childhood asthma did not indicate a role for Prevotella [34]. Other research linking Prevotella and asthma focuses on the pulmonary microbiome. Although its role in the lung is still ill-defined, evidence was provided that the reduced presence of Prevotella in the lungs of asthma and COPD patients is a result of and not a risk factor for disease progression [28].

SCFAs are considered the bridge between gut fermentation and the immune system [35]. They enter colonocytes using a range of transporters in a hydrogen- or sodium-dependent manner. Here, they are either consumed by the epithelial cells or enter systemic circulation via both active and passive transporters [36]. SCFAs can exert their role as signaling molecules, for example, by binding G-protein-coupled receptors that are found on cells throughout the whole body, including immune cells. SCFAs are associated with binding to GPR43, GPR41 and GPR109A. Upon binding, downstream effector pathways can be affected, such as the activation of mitogen-activated protein (MAP) kinases, phosphoinositide 3 (PI3K) kinases, mammalian target of rapamycin (mTOR) and pathways involving intracellular β-arrestins [37]. All these pathways are associated with inflammatory responses. Additionally, SCFAs can act as histone deacetylase (HDAC) inhibitors, thereby changing the chromatin structure and consequently the gene transcription of cells. Epigenetic studies have associated several epigenetic changes, e.g., in Th2-cells, with allergic disease outcomes, opening the door to a potential beneficial role of SCFAs on these changes [38,39,40,41,42,43,44]. Despite the FOS1:1-induced increase in *Prevotellaceae* in the gut, which has the potency to ferment FOS into SCFAs [26], there were no differences in the cecal SCFA levels between HDM-exposed control mice and HDM-exposed mice receiving any of the FOS interventions. As explained above, a possible discrepancy in the location of fermentation in the gut might underly the different effects on the microbiome. However, as multiple bacterial species can ferment different types of fibers into acetate, and bacteria can also cross-feed with acetate, a difference in microbial abundance or acetate production does not necessarily lead to a difference in acetate concentrations [45]. Interestingly, in the serum and lung of mice that received the FOS diets, increased acetate levels were found compared to HDM-exposed control mice, indicating a restoration of the acetate levels to those found in sham mice. We described previously that HDM-exposed mice show decreased serum and lung acetate levels, and that these correlate negatively with type 2 allergic markers [15]. Although the cause of this effect is yet unknown, hypotheses include HDM-induced microbiome changes that affect the expression of transporters that carry acetate across the colonic epithelium and the use of acetate as an energy source by immune cells locally in the lung tissue [15]. It remains to be studied how the FOS1:1 and FOS9:1 diets lead to a restoration of these acetate levels, but based on the mentioned hypotheses, the effects of SCFA transporters by the altered microbiome could play a role. Alternatively, locally in the lung tissue, the acetate levels may fluctuate depending on immune activation and metabolism and the serum levels may be a reflection of this.

Acetate previously altered the gene expression of immune-modifying peptides, thereby promoting Treg differentiation, leading to the suppression of allergic airway disease [46]. However, in our study, the dietary interventions did not appear to induce changes in the Treg population. As intestinal production of acetate was not influenced by the diet, the difference in the systemic acetate levels can possibly be explained by the altered systemic uptake or systemic consumption of acetate. Acetate could be a possible energy source for immune cells, such as M2 macrophages and memory T-cells via fatty acid oxidation [47]. We hypothesize that the fermentation products of FOS1:1 and FOS9:1, other than SCFAs, play a role in modifying the immune responses. One such fermentation product might be indole-3-propionic acid (IPA), which was found to protect against airway inflammation in antibiotic-treated HDM-allergic mice [48]. Interestingly, IPA was also shown to reduce fatty acid oxidation in cardiomyocyte cell lines [49], which might relate to the increased systemic acetate levels. Further research is needed to explore such a hypothesis.

Airway resistance was increased at baseline in HDM-allergic mice compared to sham. Intake of the FOS1:1, but not the FOS9:1, diet by HDM-exposed mice significantly prevented this increase in baseline airway resistance. FOS diets did not affect airway resistance when mice were exposed to methacholine. Other dietary fiber intervention studies in mice, aiming to improve airway resistance, had very diverse success rates [11,46,50,51,52,53]. Airway remodeling is a complex process, involving the airway epithelium, epithelial cytokines, downstream immune cell actions and genetic and epigenetic changes in cells [54], leaving many options for intervention. The differences in fiber type, fiber dose and the duration of the dietary intervention likely explain the diverse success rates of the multiple studies aiming to improve airway function. Nevertheless, our study indicates that FOS1:1 has a slightly better effect in protecting against an HDM-induced rise in basal airway resistance compared to FOS9:1. In future studies, lung histology should be included to provide further insight into whether FOS supplementation influences the features of airway remodeling.

In addition to the functional airway resistance measurements, we also investigated several inflammatory parameters. FOS diets did not prevent the inflammatory response of the mice to the HDM allergen, as we observed no changes in eosinophil influx in the BALF, nor in the systemic allergen-specific IgE levels (although FOS9:1 did decrease total IgE). Nevertheless, the frequency and functionality of immune cells, specifically T-cells, in the lungs were altered in the HDM-allergic mice that received 1% (*w*/*w*) FOS1:1. Previous studies with the same experimental set-up showed that the addition of 1% (*w*/*w*) fructo-oligosaccharides (1:1 ratio) combined with *B. breve* [11] or 1% galacto-oligosaccharides [50] was able to decrease the total cell influx in the BALF, including eosinophils. Also, studies with much higher doses of fiber supplementation (30–63%) were successful in lowering cellular influx in the BALF, including eosinophils [46,52,53]. In these studies, the diets induced a more robust increase in SCFA levels than in our study. We therefore hypothesize that in this study, FOS1:1 modulated immune polarization and lowered type 2 inflammation, but was not strong enough to fully block the generation of HDM-IgE synthesis during HDM sensitization and repeated HDM challenges.

When mice were exposed to HDM, it was observed that propionate supplementation via drinking water did not lower the proportion of CD4+ T-cells, but did decrease the activation state of these cells in the lung-draining lymph nodes. Researchers showed that this phenomenon was associated with reduced maturation of dendritic cells in the bone marrow due to SCFA exposure, leading to ineffective activation of Th2-cell response [52].

We observed an increase in Th17-cells in the lungs of HDM-allergic mice, but FOS diets did not affect this increase. Th17-cells, and their cytokine and chemokine secretions, have been associated with increased disease severity and airway resistance in asthma [2]. Although co-production of IL-17 and Th2 cytokines was observed in asthma patients and linked to disease exacerbation [55], Th17 differentiation is in general less associated with T2-high asthma, which we aimed to model, and more with T2-low asthma, characterized by neutrophil recruitment and activation [2]. However, our murine model showed the typical type 2 inflammation and eosinophilic airway inflammation.

Although the Th2/Th1 balance was shifted more toward Th1 in HDM-allergic mice that received an FOS1:1 diet, this was not reflected by the cytokines measured in the lung homogenates. The activity of Th2-cells, characterized by the CD69+ marker on Th2-cells, was also not altered by the diets. Nonetheless, when the lung cells of mice were re-exposed to HDM ex vivo, a significant decrease in the IL-5 concentration, and a similar pattern for IL-13, was observed in the supernatant of lung cells of HDM-allergic mice that received FOS1:1 supplementation. The FOS9:1 diet showed a similar declining pattern. This does indicate FOS is able to functionally limit type 2 responses, since in ex vivo HDM-exposed whole-cell suspensions, allergen-specific T-cells secreted fewer type 2 cytokines. Trompette et al. showed that propionate treatment in HDM-allergic mice can reduce the expression of T-cell effector/memory marker CD44 [52]. However, the propionate levels were not increased by the FOS diets in our study, nor did we observe a decrease in activation marker CD69. By contrast, the high-fiber diet (pectin 30%) was capable of decreasing type 2 cytokines in the lungs [52]. A similar result was obtained using FOS1:1 (1% *w*/*w*) in the current study, albeit only upon HDM restimulation of the lung cells, suggesting type 2 cytokine secretion of allergen-specific T-cells to be reduced. Of note, ILC2s could be an additional source of IL-5 and IL-13, but this could not be supported by our observation as these cells were not significantly increased in the BALF of HDM-allergic mice. Moreover, they remained unaltered by the dietary interventions. Intracellular cytokine staining could further confirm the type 2 lowering effects of dietary FOS1:1 in T-cells.

## 5. Conclusions

In summary, our findings indicate that dietary supplementation with FOS, before and during HDM exposure, modestly influenced the development of allergic airway inflammation by dampening type 2 cytokine release from lung lymphocytes upon allergen challenge. These effects point toward subtle immune modulation rather than established control of inflammation. The size distribution of FOS fibers did differentially affect microbiome composition, fermentation dynamics and immune features, with the 1:1 short- to long-chain FOS ratio steering away from the type 2 immune signature significantly. However, the 9:1 formulation did show a similar protective immunomodulatory pattern. While the overall impact on airway inflammation was limited, the data support a potential for structural tailoring of prebiotic fibers in fine-tuning immune responses along the gut–lung axis. Increasing the dietary dose or extending the intervention period may further enhance these effects. Collectively, these results provide a basis for future studies aimed at defining how the FOS composition and fermentation site influence microbial metabolites and downstream immune regulation in allergic airway disease.

## Data Availability

The raw data supporting the conclusions of this article will be made available by the authors on request.

## Abbreviations

The following abbreviations are used in this manuscript:

HDM	House dust mite
FOS	Fructo-oligosaccharides
BALF	Bronchoalveolar lavage fluid
SCFA	Short-chain fatty acid
*B. breve*	*Bifidobacterium breve*
i.n.	Intranasally
R_L_	Pulmonary resistance
FCS	Fetal calf serum
FMO	Fluorescence Minus One
ILC2	Innate lymphoid cells group 2
TLR	Toll-like receptor
IPA	Indole-3-propionic acid

## Appendix A. Methods

### Appendix A.1. Details on Animal Procedures—House Dust Mite (HDM) Treatment

Cages with mice were moved to a different room for procedures. Per cage, animals were placed in a plastic see-through box that was consequently filled with isoflurane gas (4% oxygen). When animals lay still, oxygen was reduced to 1.5%. Animals were taken out one by one for the PBS treatment with or without HDM. Animals were held on their back on one hand of the researcher, while closing the mouth of the mice with the thumb. With the other hand, 40 µL PBS or PBS with HDM was pipetted on the nose of the mouse using a 200 µL pipet. After the drops disappeared in the nose and two extra breathing cycles, the mouse was put back in its cage to wake up. The isoflurane box was cleaned with a paper towel in between cage treatments.

### Appendix A.2. Details on Short-Chain Fatty Acid Determination Using LC-MS/MS

**Sample and standards preparation**

Supernatant of cecum content homogenates, serum samples and supernatant of lung homogenates were analyzed. Cecum content homogenate samples were diluted in 50% (*v*/*v*) acetonitrile to fit the range of the measurements. This was not needed for the serum and lung samples. The 10 µL (diluted) sample and 20 µL internal standards dilution in acetonitrile were added to a polypropylene 96-well plate with a conical bottom. After closing with a silicone mat, the plate was vortex mixed vigorously for several seconds and afterwards centrifuged for 5 min at 2643× *g*. Next, 20 µL of the supernatant was pipetted into a polypropylene 96-deep well plate and 10 µL of 0.2 M 3-nitrophenylhydrazine hydrochloride (3-NPH) (Sigma-Aldrich) solution in 50% acetonitrile and of 10 µL 0.12 M -ethyl-3-(3-dimethylaminopropyl) carbodiimide (EDC) hydrochloride (Carl Roth) in a mixture of pyridine/acetonitrile/water (0.5/49.5/49.5% (*v*/*v*/*v*)) were added. Then, the plate was closed again with the mat and gently mixed. After this, the plate was incubated in a water bath of 40 °C for 30 min, after which the plate was put on ice. Finally, samples were diluted with 200 µL water and stored at 4 °C until analysis.

Standard samples were prepared in 50% (*v*/*v*) acetonitrile in the ranges 20–5000 µM (acetate), 1–250 µM (propionate) and 0.4–100 µM (butyrate) (Sigma-Aldrich). Quality control samples were prepared in 50% (*v*/*v*) acetonitrile at 4000; 400; 40 µM (acetate), 200; 20; 2 µM (propionate) and 80; 8; 0.8 µM (butyrate). Internal standards of acetic acid-d4 (50 µM) (Thermo-Scientific), propionic acid-d3 (20 µM) (Toronto Research Chemicals) and butyric acid-d7 (10-µM) (Cayman chemical) were prepared in acetonitrile. All these solutions were stored at 4 °C until analysis.

**Analytical instruments**

For separation, we used the Shimadzu Nexera X2 chromatographic system (Kyoto, Japan). This contained two LC30-AD pumps, an Sil30-ACmp autosampler, DGU-250 AR degasser and a CTO-20 AC column oven. For detection, we used the AB-SCIEX QTRAP^®^ 5500 triple quadrupole mass spectrometer (Concord, ON, Canada), containing a Turbo Ion™ TurboIonSpray^®^ probe and inlet valve. Analyst 1.6.2 software (Sciex, Marlborough, MA, USA) was used for data collection and instrument control. LC-MS/MS data were processed using MultiQuant 3.0.1 software (Sciex).

**LC-MS/MS conditions**

A 3 µL sample was injected on the VisionHT C18-P (50 × 2.0 mm, 1.5 µm) with a VisionHT C18 Polar guard (7.5 × 2.0 mm, 1.5 µm). The temperatures of the autosampler and column were kept at 4 °C and 60 °C, respectively. The gradient elution was performed at 0.5 mL/min with water (0.1% formic acid (*v*/*v*)) and acetonitrile (Sigma-Aldrich). Acetonitrile increased linearly from 0 to 2.5 min from 5% to 15%. During 2.51–2.80 min, the column was flushed with 100% acetonitrile. After that, the gradient went back to 5% acetonitrile and remained as such until the end of the run at 3 min. For the detection mode, multiple reaction monitoring (MRM) in negative mode was used. The parameters for detection included the following: curtain gas 20 psi, ion spray voltage −1200 V, temperature 700 °C, ion source gas (1) 60 psi, ion source gas (2) 80 psi. Further, compound-dependent parameters can be found in Appendix A Table A1.

**Table A1:** MRM transitions of SCFAs.

3-NPH Derivatized Compound	Q1 Mass (*m*/*z*)	Q3 Mass (*m*/*z*)	Declustering Potential (V)	Collision Energy (V)	Collision Cell Exit Potential (V)	Dwell Time (ms)
Acetate	195.0	153.0	−45	−20	−7	25
Propionate	208.0	165.0	−125	−18	−9	25
Butyrate	222.0	137.0	−135	−26	−9	25
Acetate-d3	197.0	137.0	−45	−26	−9	10
Propionate-d3	211.0	137.0	−125	−26	−9	10
Butyrate-d7	229.0	137.0	−135	−26	−9	10

### Appendix A.3. Details on Fecal Sample DNA Extraction and Preparation for Sequencing

DNA was extracted from samples using MagPure Stool DNA KF Kit B (MAGEN, Guangzhou, China) according to the manual’s instruction. Transfer 100–200 mg sample to the centrifuge tube with grinding beads. Add 1 mL Buffer ATL/PVP-10, grinding the sample in the grinding machine (Shanghai Jingxin Tech, Shanghai, China) and incubating at 65 °C for 20 min. The mixture was centrifuged at 14,000× *g* for 5 min (Eppendorf, Hamburg, Germany). Then, the supernatant was transferred to a new tube. Add an amount of 0.6 mL buffer PCI to the sample and then mix thoroughly by vortexing for 15 s. The mixture was centrifuged at 18,213× *g* for 10 min. The supernatant was transferred to a deep well plate with a magnetic beads binding solution (600 μL buffer with magnetic beads + 20 μL proteinase K + 5 μL RNase A, 700 μL Wash 1, 700 μL Wash 2, 700 μL Wash 3, 100 μL elution buffer). Transfer the sample to the corresponding place in the deep well plate of the machine (Kingfisher, Thermo Fisher, USA). Begin the corresponding program in Kingfisher. Transfer the DNA to a 1.5 mL centrifuge tube for storage when the program runs out.

The library was prepared by 2 × Phanta Max Master Mix (VAZYME, Nanjing, China) polymerase, and the V3V4 variable region of 16S rDNA of bacteria was amplified by forward and reverse PCR degenerate primers F and R (338F:ACTCCTACGGGAGGCAGCAG, 806R:GGACTACHVGGGTWTCTAAT). PCR enrichment was performed in a 50 μL reaction containing 30 ng template and fusion PCR primers. PCR cycling conditions were as follows: 95 °C for 3 min; 30 cycles of 95 °C for 15 s, 56 °C for 15 s, 72 °C for 45 s and final extension at 72 °C for 5 min. PCR products were purified by DNA magnetic beads (BGI, LB00V60).

Next, the final double-strand library products are denatured to generate the single-strand library products. Then, the circularization reaction is set up to obtain single-strand circularized DNA products. Any single-strand linear DNA will be digested to remove. The final single-strand circularized library is amplified with phi29 and rolling circle amplification (RCA) to generate the DNA nano ball (DNB), which carries multiple copies of the initial single-stranded library molecule. The DNBs are loaded into the patterned nanoarray and sequencing reads of PE300 base length are generated with DNBSEQ-G400 platform (BGI-Shenzhen, China).

### Appendix A.4. Detailed Information on Antibodies Used for Flow Cytometry

**Table A2:** Detailed information on antibodies for flow cytometry.

Marker	Fluorochrome	Clone	Company
BALF general differentiation			
Fixable Viability Dye	APC-Cy7	n.a.	Thermo Fisher Scientific
CD3e	PE-Cy7	145-2C11	Thermo Fisher Scientific
MHCII (I-A)	FITC	NIMR-4	Thermo Fisher Scientific
CD11c	APC	N418	Thermo Fisher Scientific
B220 (CD45R)	PE-Cy7	RA3-6B2	Thermo Fisher Scientific
CCR3	PE	J073E5	BioLegend
BALF ILC2			
CD3	PE	145-2c11	eBioscience
CD5	PE	53-7.3	eBioscience
CD8a	PE	53-6.7	eBioscience
CD11b	PE	M1/70	eBioscience
CD11c	PE	N418	eBioscience
CD19	PE	1D3	Becton Dickinson, Franklin Lakes, NJ, USA
B220	PE	RA3-6B2	Becton Dickinson
FceRIa	PE	MAR-1	eBioscience
NK1.1	PE	Pk136	eBioscience
Ter-119	PE	TER-119	eBioscience
Gr-1	PE	RB6-8C5	Becton Dickinson
CD90.2	FITC	53-2.1	Becton Dickinson
KLRG1	PE CF594	2F1	Becton Dickinson
CD25	Percp cy5.5	PC61.5	eBioscience
ICOS	PE Cy7	7E.17G9	eBioscience
CD127	EF450	A7R34	eBioscience
Aqua LD	Amcyan	n.a.	-
CD4	BV605	RM4-5	Becton Dickinson
CD117	BV650	2B8	Becton Dickinson
Sca-1	BV786	D7	Becton Dickinson
Gata3	APC	TWAJ	eBioscience
Ki-67	AF700	SolA15	eBioscience
T1ST2	Bio	DJ8	MDBioproducts
Lung Th1-Th2			
Fixable Viability Dye	APC-Cy7	n.a.	Thermo Fisher Scientific
CD4	BV510	RM4-5	BioLegend
CD69	PE-Cy7	H1.2F3	Thermo Fisher Scientific
CXCR3	PE	CXCR3-173	Thermo Fisher Scientific
T1ST2 (IL-33R)	FITC	DJ8	MDBioproducts
Fixable Viability Dye	APC-Cy7	n.a.	Thermo Fisher Scientific
CD4	BV510	RM4-5	BioLegend
CD25	PerCP-Cy5.5	PC61.5	Thermo Fisher Scientific
CD127	PE-Cy7	eBioSB/199 (SB/199)	Thermo Fisher Scientific
Foxp3	FITC	FJK-16s	Thermo Fisher Scientific
Roryt	APC-Cy7	Q31-378	BD Biosciences
CD196 (CCR6)	PE	29-2L17	BioLegend
Lung Th17-Treg			
Fixable Viability Dye	APC-Cy7	n.a.	Thermo Fisher Scientific
CD3e	PE-Cy7	145-2C11	Thermo Fisher Scientific
MHCII (I-A)	FITC	NIMR-4	Thermo Fisher Scientific
CD11c	APC	N418	Thermo Fisher Scientific
B220 (CD45R)	PE-Cy7	RA3-6B2	Thermo Fisher Scientific
CCR3	PE	J073E5	BioLegend
CD3	PE	145-2c11	eBioscience
CD5	PE	53-7.3	eBioscience
CD8a	PE	53-6.7	eBioscience
CD11b	PE	M1/70	eBioscience
CD11c	PE	N418	eBioscience
CD19	PE	1D3	Becton Dickinson
B220	PE	RA3-6B2	Becton Dickinson
FceRIa	PE	MAR-1	eBioscience
NK1.1	PE	Pk136	eBioscience
Ter-119	PE	TER-119	eBioscience
Gr-1	PE	RB6-8C5	Becton Dickinson
CD90.2	FITC	53-2.1	Becton Dickinson
KLRG1	PE CF594	2F1	Becton Dickinson
CD25	Percp cy5.5	PC61.5	eBioscience
ICOS	PE Cy7	7E.17G9	eBioscience
CD127	EF450	A7R34	eBioscience

## Appendix B

**Table A3:** Nutritional composition of the dietary interventions per intervention group.

		AIN93-G	AIN93-G	AIN93-G	AIN93-G	AIN93-G	AIN93-G
g/1 kg Feed		Control	1% scFOS/lcFOS 9:1	1% scFOS/lcFOS 1:1	1% scFOS/lcFOS 1:9 + 2% Probiotics	1% scFOS/lcFOS 1:1 + 2% Probiotics	2% Probiotics
Functional		g/kg Feed	g/kg Feed	g/kg Feed	g/kg Feed	g/kg Feed	g/kg Feed
**Carbs**	**Raw Material names**						
cornstarch	C gel 03401	397.49	397.49	397.49	377.49	377.49	377.49
dextrinized cornstarch	Maltodextrin MD1925 WS	132.0	131.3	131.6	131.3	131.6	132.0
sucrose	Sugar Melis	100.0	100.0	100.0	100.0	100.0	100.0
**Fiber**							
fiber source (cellulose)	Arbocel B800	50.00	39.95	39.95	39.95	39.95	50.00
Inulin HP (lcFOS) (97% FOS fiber)	Inulin Fiber	0.0	1.0	5.2	1.0	5.2	0.0
Raftilose P95 (scFOS) 90% FOS	Oligofructose Powder	0.0	10.0	5.6	10.0	5.6	0.0
**Beneficial microbes**							
*B. breve M16-V*		0.00	0.00	0.00	20.00	20.00	20.00
**Protein**							
Soy protein	Soy protein isolate	200.0	200.0	200.0	200.0	200.0	200.0
DL-methionine	DL-Methionine	2.0	2.0	2.0	2.0	2.0	2.0
L-cystine	L-Cystine	1.0	1.0	1.0	1.0	1.0	1.0
**Fat**							
soybean oil	Soy oil non-GMO	70.0	70.0	70.0	70.0	70.0	70.0
**Others**							
Mineral mix	Mineral mix AIN 93G	35.0	35.0	35.0	35.0	35.0	35.0
Vitamin mix	Vitamin mix AIN 93VX	10.0	10.0	10.0	10.0	10.0	10.0
Choline bitartrate	Choline bitartrate	2.50	2.50	2.50	2.50	2.50	2.50
TBHQ	tert-butylhydroquinone	0.014	0.014	0.014	0.014	0.014	0.014
**Nutritional value**							
	Total weight	1000.0	1000.2	1000.3	1000.2	1000.3	1000.0
	Dry mass	932.9	932.9	932.9	932.9	932.9	932.9
	Protein	180.0	180.0	180.0	180.0	180.0	180.0
	Total Carbohydrates	592.0	592.0	592.0	592.0	592.0	592.0
	Fat	72.0	72.0	72.0	72.0	72.0	72.0
	Dietary Fiber	49.8	49.8	49.8	49.8	49.8	49.8
	Kcal	3835.4	3835.4	3835.4	3835.4	3835.4	3835.4
